# Weight Gain After Smoking Cessation and Risk of Major Chronic Diseases and Mortality

**DOI:** 10.1001/jamanetworkopen.2021.7044

**Published:** 2021-04-27

**Authors:** Berhe W. Sahle, Wen Chen, Lal B. Rawal, Andre M. N. Renzaho

**Affiliations:** 1 School of Social Sciences and Psychology, Western Sydney University, Sydney, Australia; 2 Melbourne School of Population and Global Health, University of Melbourne, Melbourne, Australia; 3 Department of Medical Statistics, School of Public Health and Center for Migrant Health Policy, Sun Yat-sen University, Guangzhou, China; 4 Center for Migrant Health Policy, Sun Yat-sen University, Guangzhou, China; 5 School of Health, Medical and Applied Sciences, Central Queensland University, Sydney, Australia; 6 Translational Health Research Institute, School of Medicine, Western Sydney University, Sydney, Australia

## Abstract

**Importance:**

Smoking cessation is frequently followed by weight gain; however, whether weight gain after quitting reduces the health benefits of quitting is unclear.

**Objective:**

To examine the association between weight change after smoking cessation and the risk of cardiovascular diseases (CVD), type 2 diabetes, cancer, chronic obstructive pulmonary disease (COPD), and all-cause mortality.

**Design, Setting, and Participants:**

This cohort study analyzed data from a nationally representative sample of Australian adults aged 18 years or older who were studied between 2006 and 2014. Smoking status and anthropometric measurements were self-reported annually. Cox proportional hazards regressions were used to determine the hazard ratios (HRs) for the association between changes in weight and body mass index (BMI) and the risk of CVD, type 2 diabetes, cancer, COPD, and mortality. Data were analyzed in January 2019.

**Exposures:**

Annual self-reported smoking status; years since quitting.

**Main Outcomes and Measures:**

Weight gain after quitting, incident CVD, type 2 diabetes, cancer, COPD, and all-cause mortality.

**Results:**

Of a total 16 663 participants (8082 men and 8581 women; mean [SD] age, 43.7 [16.3] years), those who quit smoking had greater increases in weight (mean difference [MD], 3.14 kg; 95% CI, 1.39-4.87) and BMI (MD, 0.82; 95% CI, 0.21-1.44) than continuing smokers. Compared with continuing smokers, the HRs for death were 0.50 (95% CI, 0.36-0.68) among quitters who lost weight, 0.79 (95% CI, 0.51-0.98) among quitters without weight change, 0.33 (95% CI, 0.21-0.51) among quitters who gained 0.1 to 5.0 kg, 0.24 (95% CI, 0.11-0.53) among quitters who gained 5.1 to 10 kg, and 0.36 (95% CI, 0.16-0.82) among quitters who gained more than 10 kg. The HRs for death were 0.61 (95% CI, 0.45-0.83) among quitters who lost BMI, 0.86 (95% CI, 0.51-1.44) among quitters without change in BMI, 0.32 (95% CI, 0.21-0.50) among quitters who gained up to 2 in BMI, and 0.26 (95% CI, 0.16-0.45) among quitters who gained more than 2 in BMI.

**Conclusions and Relevance:**

This cohort study found that smoking cessation was accompanied by a substantial weight gain; however, this was not associated with an increased risk of chronic diseases or an attenuation of the mortality benefit of cessation.

## Key Points

**Question:**

Is weight gain following smoking cessation associated with an increase in the risk of chronic diseases or mortality?

**Findings:**

In this nationally representative cohort study of 16 663 Australian adults studied between 2006 and 2014, people who quit smoking had significantly lower risk of death than those who continued smoking regardless of change to weight and body mass index after quitting. Neither weight change nor body mass index change following smoking cessation was significantly associated with the risk of cardiovascular disease, type 2 diabetes, cancer, or chronic obstructive pulmonary disease.

**Meaning:**

These findings suggest that postcessation weight gain poses a trivial risk of harm compared with the benefits of quitting, and smoking cessation interventions should include messages about the safety of postcessation weight gain.

## Teaser

This cohort study using a nationally representative database of Australian adults examines the association of weight gain following smoking cessation with risk of cardiovascular disease, type 2 diabetes, cancer, or chronic obstructive pulmonary disease.

## Introduction

Smoking cessation reduces the risk of major chronic diseases,^1^ increases life expectancy,^1,2^ and improves quality of life.^2,3^ Despite these significant benefits, smoking cessation is often accompanied by increases in weight and body mass index (BMI; calculated as weight in kilograms divided by height in meters squared).^4,5,6^ Several mechanisms have been proposed to explain weight gain after smoking cessation, including removal of the appetite-suppressant effects of nicotine, leading to decreased metabolic rate and increased energy expenditure.^7,8^ Previous cohort studies^5,9,10^ and meta-analyses of observational studies^4,6^ have established that smokers who quit gain significantly more weight and BMI than continuing smokers, although there is considerable variability in the amount of weight gain after quitting and it may affect health.

Excess weight is an established risk factor for several health problems, including type 2 diabetes, cardiovascular diseases (CVD), some cancers, chronic obstructive pulmonary diseases (COPD), and mortality.^11,12,13,14,15^ In a study including more than 10 million participants from 239 prospective studies, all-cause mortality was minimal for persons with BMI between 20 and 25; however, each 5-unit increase in BMI above 25 was associated with a 49%, 38%, and 19% higher risk of CVD, respiratory disease, and cancer mortality, respectively.^13^ Another study based on data from 12 European cohort studies found that each 5-unit increase in BMI was associated with an increase in CVD mortality of 34% in men and 29% in women.^16^ Therefore, there is a concern that weight or BMI gain after smoking cessation could increase the risk of chronic diseases and potentially attenuate the benefits of quitting smoking.^4,5,17^ Furthermore, among smokers, concern about weight gain after smoking cessation is a frequently cited barrier for not trying to quit or for relapsing after quitting attempts.^18^

Whether the health risks in the community caused by weight or BMI gain after smoking cessation exceed the protective benefits of quitting smoking is not conclusive. Previous studies assessing the health risks associated with weight or BMI gain after smoking cessation have only been limited to CVD or type 2 diabetes,^5,9,10,19^ and the association with other chronic diseases potentially associated with smoking and weight gain, such as COPD and cancers, remains unknown. For example, a 2018 study by Hu et al^5^ provides important information regarding postcessation weight gain and its association with chronic conditions and mortality. However, the study used a cohort of health workers and included only a few health outcomes (ie, CVD, type 2 diabetes, and mortality). Therefore, whether the findings from this study apply to other conditions and to the general population is still unknown. Additionally, the existing scant evidence on the association between weight or BMI gain after quitting smoking and the risk of type 2 diabetes remains inconsistent as well, with some studies showing a significant decrease^5,10,19^ while others report a significant increase^5,20^ in the risk of type 2 diabetes. A significant decrease in mortality has also been reported,^5^ while other studies suggest no significant associations between gaining 5 kg or more in weight after smoking cessation and the risk of CVD^9^ or mortality.^19^ Possible reasons for these discrepant findings may include variation in duration of follow-up after smoking cessation^5,9,17^ and the use of smoking-cessation therapies and interventions to prevent weight gain.^7,17^

A better understanding of the associations between long-term weight and BMI change following smoking cessation and a range of health outcomes might facilitate smoking cessation and inform the design of interventions to reduce excess health risks among adults quitting smoking. This nationally representative longitudinal study used a cohort of 16 663 Australian adults to estimate weight and BMI gain following smoking cessation and its association with the risk of CVD, type 2 diabetes, cancer, COPD, and mortality over time.

## Methods

### Data Source and Participants

This study used data from the Household, Income and Labour Dynamics in Australia (HILDA) survey, a panel survey that commenced in 2001 (wave 1) and includes a national probability sample of 19 914 individuals residing in 7682 Australian households. All Australian states and territories were included with households selected from 488 census districts across Australia. Interviews are conducted annually with all adult members of each household. A further 5451 individuals (2153 households) were added to the sample in wave 11 as part of a general top-up to retain cross-sectional representativeness in the sample. Wave 1 represents all persons residing in private dwellings in Australia, which constitutes the reference population. The response rate in each study wave was reasonably high, ranging from a low of 66.5% in wave 14 to a high of 74% in wave 7. Details on the study design, study procedures, and participants have been described elsewhere.^11,21^

The present study covers study waves between 2006 (wave 6) to 2014 (wave 13) because questions on long-term chronic conditions were introduced in wave 3 and anthropometric measurements in wave 6. Participants with prevalent CVD, type 2 diabetes, cancer, or COPD prior at wave 3 were excluded from the analyses to minimize the probability that people had quit smoking as a consequence of those diseases. We restricted our analysis to participants aged 18 years and over with nonmissing values on smoking status, weight, and BMI. This is because it is illegal to sell tobacco products to people under the age of 18 in most Australian states and territories^22^ and we did not expect a significant risk of development of the chronic diseases of interest in a 10-year time frame for an 18-year-old.

### Assessment of Smoking and Smoking Cessation

At every wave of the HILDA survey, participants reported whether they were smokers, never smoked, or quit smoking in the previous 12 months. Past and continuing smokers were asked whether they had smoked at least 100 cigarettes in their entire life. We defined people quitting smoking as those who reported having stopped smoking and did not report being continuing smokers at any of the subsequent waves. Those who reported being past smokers in one wave but being continuing smokers in the subsequent survey were considered continuing smokers. Duration of smoking cessation was estimated as the difference between date of onset of quitting and the last follow-up date.

### Assessment of Weight and BMI Change

The HILDA survey participants returning the self-completion questionnaire reported on their height and weight. Self-reported height and weight were used to derive BMI for each participant. The distribution of BMI scores in the HILDA survey compares reasonably well with the BMI data for Australians collected by the Australian Bureau of Statistics.^23^ We calculated weight change after smoking cessation as the difference in body weight recorded at the wave when quitting was reported and weight recorded at the last follow-up. We applied the cut-off values used in previous studies to classify people as those who lost weight, had no weight change, or had weight gain of 0.1 to 5.0 kg, 5.1 to 10.0 kg, or more than 10.0 kg.^5^ We used the same approach to estimate change in BMI, and categorized participants as those who lost BMI, had no change in BMI, gained 0.1 to 2.0, or gained more than 2.

### Assessment of Health Outcomes

In waves 3, 7, 9, and 13, participants were asked the following question: “Have you ever been told by a doctor or nurse that you have any of the following long-term health conditions? Please only include those conditions that have lasted or are likely to last for 6 months or more.” Responses included CVD (eg, heart disease, circulatory diseases), COPD, type 2 diabetes, and cancers. The HILDA survey has been linked to the National Death Index in 2014, to provide information on death.

### Assessment and Definition of Covariates

Participants reported on recent sociodemographic information, including age, sex, employment status, marital status, and education. The HILDA uses the Australian Bureau of Statistics Socio-Economic Indexes for Areas (SEIFA) to categorize participants into an index of socioeconomic disadvantage, where higher SEIFA scores indicate a relative lack of disadvantage in general.^11^

Information on physical activity was collected across all waves by asking the frequency of participation in regular moderate or intense physical activity for at least 30 minutes in a week. This single item is commonly used in the literature to assess physical activity.^24^ Six precoded responses ranging from “not at all” to “every day” were offered, which were in turn categorized into not at all or less than once a week, 1 to 3 times a week, and 4 or more times a week.^25^

In the HILDA survey, respondents were asked how often in a week they regularly consume alcohol and provided with precoded response categories ranging from never to every day. Current drinkers reported on the number of standard drinks (with 1 standard drink estimated to contain 10 g of alcohol) consumed on a usual drinking occasion. Level of alcohol consumption was defined according to the National Institute on Alcohol Abuse and Alcoholism (NIAAA).^26^ According to the NIAAA, for women, low-risk drinking is defined as no more than 3 drinks on any single day and no more than 7 drinks per week. For men, it is defined as no more than 4 drinks on any single day and no more than 14 drinks per week.^26^

Dietary intake was assessed using a 12-item food frequency questionnaire that asked participants about the frequency (ie, ≥2 per day, daily, 2-6 times per week, once per week, 1-3 times a month, and never) of their usual consumption of 12 food types. Each food item in the list is classified as a “core healthy” or “discretionary” food.^27^ For each food type, consumption of at least once per week was considered as an average daily intake. Responses for regular consumption (yes = 1 and no = 0) for each type of food were used to calculate a mean score of consumption of core and discretionary foods in the study sample. Responding participants were then categorized as having “lower-than-average daily intake of core foods” or “higher-than-average daily intake of discretionary foods.”^27^ The HILDA survey participants also reported whether they had been dieting to lose weight.

### Statistical Analyses

Descriptive statistics at wave 6 (baseline) are presented as means (with SDs) for continuous variables and percentages for categorical variables. Multiple linear regression analysis was used to assess weight and BMI changes according to smoking status and duration of smoking cessation. Cox proportional hazards regressions were used to determine the hazard ratios (HRs) along with 95% CIs for the association between weight and BMI change and the risk of CVD, type 2 diabetes, cancer, COPD, and all-cause mortality. In the Cox regression models, we compared the risk of CVD, type 2 diabetes, cancer, COPD, and all-cause mortality between: (1) smokers who quit vs continuing smokers, (2) smokers who quit vs never smoked, (3) smokers who quit with weight change vs smokers who quit without weight change, and (4) smokers who quit with BMI change vs smokers who quit without BMI change. There was a significant interaction (*P* < .001) between weight gain and duration of smoking cessation; therefore, an interaction term (the product of the quadratic term of duration and smoking status) was included in the models. We tested the proportional hazards assumption using Schoenfeld residuals after fitting Cox proportional hazards models, and the proportionality assumption was not violated. We used multiple imputation by chained equations (with 50 estimates) to impute missing values of covariates at follow-up. We used Stata version 15.0 (StataCorp LLC) for the data analyses. We used secondary data from the HILDA survey, which has been approved by the human research ethics committee of the University of Melbourne. *P* < .05 was considered significant in 2-sided tests.

## Results

Table 1 presents a summary of participant characteristics. A total of 16 663 participants aged 18 years or older constituted the study cohort (9862 men [48.5%]; mean [SD] age, 43.7 [16.3] years). Throughout the study, 3588 (21.5%) of the participants continued to smoke, 7842 (47.1%) remained as never smokers, and 5233 (31.4%) reported that they had quit smoking. Compared with the participants who continued to smoke, participants who quit smoking were less likely to be high-risk drinkers (358 [5.8%] vs 474 [10.8%]) and score in the lowest deciles of SEIFA (397 [13.5%] vs 353 [7.4%]) but more frequently consumed above average amounts of both discretionary (2341 [31.3%] vs 1741 [23.3%]) and core foods (614 [35.6%] vs 304 [17.6%]).

**Table 1.** Characteristics of Study Participants at Baseline^a^

Characteristics	Participants, No. (%)					
	Never (n = 7842 [47.1%])	Continuing (n = 3588 [21.5%])	Quitting (n = 5233 [31.4%])	Duration since quitting among those who quit		
				≤2.0 y (n = 607 [11.8%])	2.1-6.0 y (n = 1840 [35.8%])	>6 y (n = 2697 [52.4%])
Weight change, mean (SD), kg	1.96 (7.8)	1.70 (8.6)	1.48 (8.2)	0.70 (7.7)	1.37 (8.0)	2.2 (9.6)
Weight change						
Lost weight	1999 (25.5)	891 (24.8)	1517 (29.0)	154 (25.4)	554 (30.1)	785 (29.1)
No change	1997 (25.5)	1241 (34.6)	1298 (24.8)	102 (16.8)	191 (10.4)	208 (7.7)
0.1-5 kg	2190 (27.9)	690 (19.2)	1330 (25.4)	165 (27.2)	489 (26.6)	654 (24.2)
5.1-10.0 kg	976 (12.4)	399 (11.1)	629 (12.0)	34 (5.6)	212 (11.5)	377 (14.0)
≥10 kg	680 (8.7)	367 (10.2)	459 (8.8)	29 (4.8)	131 (7.1)	293 (10.9)
BMI change, mean (SD)	0.68 (2.9)	0.66 (3.3)	0.56 (3.11)	0.30 (2.7)	0.42 (3.3)	0.90 (3.5)
BMI change						
Lose weight	2360 (29.0)	1010 (27.9)	1709 (31.2)	203 (40.0)	634 (38.4)	865 (32.1)
No change	1665 (20.5)	1065 (29.4)	1119 (20.4)	67 (13.2)	122 (7.4)	123 (4.6)
0.1-2.0	2320 (28.6)	731 (20.2)	1412 (25.8)	161 (31.7)	500 (30.3)	749 (27.8)
≥2.0	1781 (21.9)	820 (22.6)	1232 (22.5)	77 (15.2)	395 (23.9)	759 (28.1)
Age, mean (SD), y	42.7 (18.1)	36.0 (14.3)	47.3 (17.5)	45.5 (20.9)	48.3 (17.9)	48.4 (14.9)
Sex						
Men	4080 (41.8)	2507 (55.1)	3275 (51.2)	318 (51.5)	989 (53.1)	1343 (49.6)
Women	5670 (58.2)	2041 (44.9)	3116 (48.4)	300 (48.5)	872 (46.9)	1367 (50.4)
SEIFA decile						
Lowest	473 (6.7)	397 (13.5)	353 (7.4)	22 (6.7)	111 (7.9)	181 (7.1)
Fifth	704 (9.9)	325 (11.1)	507 (10.6)	39 (11.9)	154 (11.0)	270 (10.6)
Top	804 (11.3)	133 (4.5)	465 (9.7)	30 (9.1)	141 (10.1)	262 (10.2)
Employment						
Employed	6067 (64.1)	2694 (61.1)	3528 (57.1)	348 (60.8)	1029 (57.4)	1419 (52.7)
Unemployed	281 (3.0)	365 (8.3)	188 (3.0)	22 (3.8)	48 (2.7)	50 (1.9)
Not in labor force	3113 (32.9)	1347 (30.6)	2466 (39.9)	202 (35.3)	715 (39.9)	1225 (45.4)
Alcohol consumption^b^						
None	1548 (16.2)	279 (6.4)	348 (5.6)	46 (7.7)	91 (5.0)	136 (5.1)
Low	7758 (81.4)	3618 (82.8)	5450 (88.5)	515 (86.7)	1620 (89.6)	2346 (88.7)
High	222 (2.3)	474 (10.8)	358 (5.8)	33 (5.6)	98 (5.4)	162 (6.1)
Physical activity, time/wk						
Not at all or <1	2336 (28.3)	1654 (30.4)	1229 (33.6)	125 (29.8)	492 (30.2)	818 (31.2)
1-3	3334 (40.3)	2028 (37.2)	1313 (35.9)	154 (36.7)	613 (37.6)	965 (36.8)
≥4	2594 (31.4)	1763 (32.4)	1111 (30.4)	141 (33.6)	524 (32.2)	840 (32.0)
Dieting to lose weight	2693 (35.3)	943 (28.7)	2046 (37.6)	157 (32.6)	657 (37.2)	1048 (38.9)
Dietary pattern						
Discretionary	3404 (45.5)	1741 (23.3)	2341 (31.3)	219 (60.2)	708 (53.5)	1176 (50.7)
Core food	806 (46.7)	304 (17.6)	614 (35.6)	48 (30.8)	187 (28.3)	340 (27.7)

Abbreviations: BMI, body mass index (calculated as weight in kilograms divided by height in meters squared); SEIFA, Socio-Economic Indexes for Areas.

^a^ Age, sex, SEIFA, employment, alcohol consumption, physical activity, and dietary pattern are at wave 6, the baseline for this study.

^b^ Categories for low- and high-risk drinking are based on National Institute on Alcohol Abuse and Alcoholism guidelines. For women, low-risk drinking is defined as no more than 3 drinks on any single day and no more than 7 drinks per week. For men, it is defined as no more than 4 drinks on any single day and no more than 14 drinks per week.

### Smoking Cessation and Weight and BMI Change

The mean (SD) unadjusted weight and BMI gain during a mean (SD) of 6.0 (3.7) years of follow-up since smoking cessation were 1.58 (7.2) kg and 0.58 (2.9), respectively. Of those who quit smoking and gained 10 kg or more, 29 (6.4%) gained in 2 or less years, 131 (28.9%) gained in 2.1 to 6.0 years, and 293 (64.1%) gained more than 10 kg weight during the first 2 years, 2 to 6 years, and after 6 years since quitting, respectively. The corresponding BMI gain of more than 2 (out of a total of 1232 participants) was 77 (15.2%), 395 (23.9%), and 759 (28.1%) participants (Table 1). Weight gain peaked between 6 to 8 years after quitting and then gradually decreased thereafter (Figure). The patterns were similar for BMI, which peaked around 8 years after smoking cessation (Figure).

**Figure.**
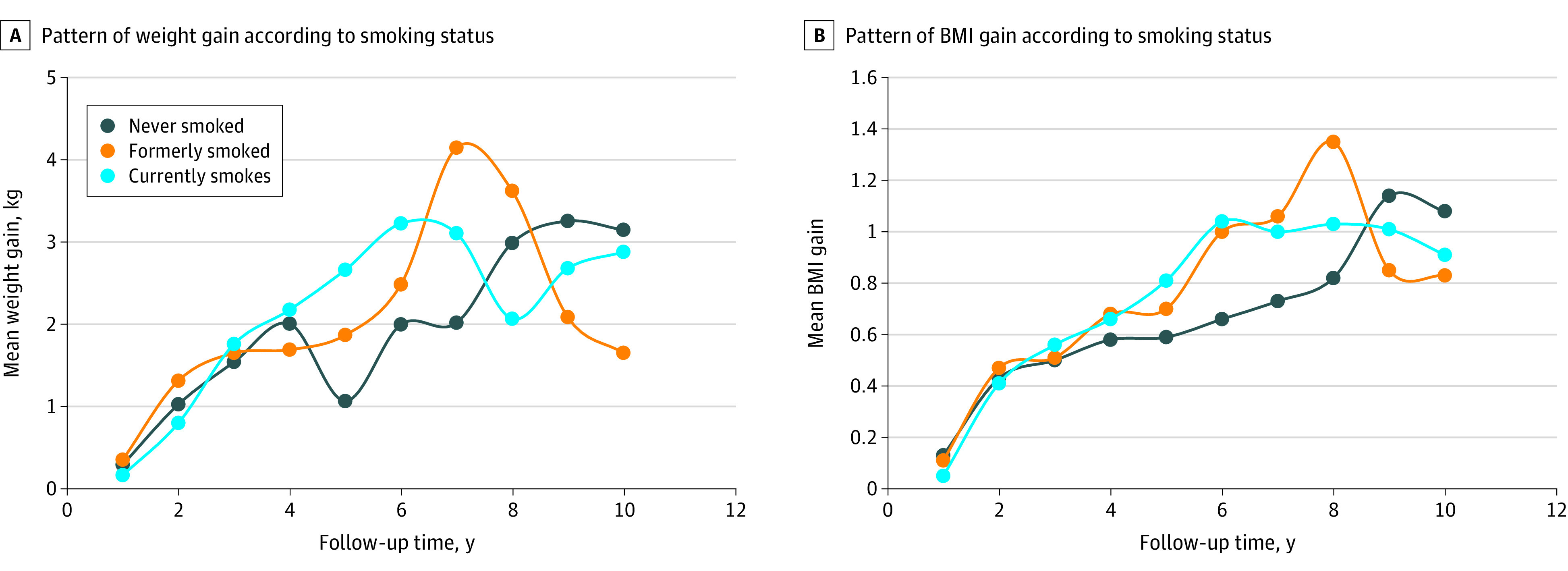
Pattern of Weight Gain and Body Mass Index (BMI) According to Smoking Status

With analyses adjusted for confounding, those who quit smoking had significantly greater weight and BMI gain compared with continuing smokers (mean difference [MD], 3.14 kg; 95% CI, 1.39 to 4.87 kg; BMI MD, 0.82; 95% CI, 0.21 to 1.44) (Table 2). The duration of smoking cessation modified the amount of weight gain (β for quadratic interaction term, −0.03; *P* = .005) and BMI gain (β for quadratic interaction term, −0.01; *P* = .02) after quitting. The difference in weight or BMI change after quitting were not modified by sex, dieting to lose weight, or whether participants had smoked at least 100 cigarettes in their lifetime or not (Table 2).

**Table 2.** Association Between Smoking Cessation and Weight and BMI Change^a^

	Weight change		BMI change	
	β (95% CI)	*P* value	β (95% CI)	*P* value
Smoking status				
Continuing	1 [Reference]	[Reference]	1 [Reference]	[Reference]
Never smoked	1.66 (−0.28 to 3.61)	.09	0.48 (−0.16 to 1.13)	.14
Former	3.14 (1.39 to 4.87)	<.001	0.82 (0.21 to 1.44)	.009
Quadratic interaction term				
Never smoked × duration	−0.02 (−0.04 to 00)	.14	−0.01 (−0.015 to 0.00)	.07
Former smoker × duration	−0.03 (−0.05 to −0.01)	.005	−0.01 (−0.02 to −0.001)	.02
Former smokers only, y since quitting				
≤2.0	1 [Reference]	[Reference]	1 [Reference]	[Reference]
2.1-6.0	1.30 (−0.10 to 2.71)	.07	0.86 (0.53 to 1.20)	<.001
>6	2.17 (0.81 to 3.52)	.002	1.24 (0.92 to 1.57)	<.001

Abbreviations: BMI, body mass index (calculated as weight in kilograms divided by height in meters squared); SEIFA, Socio-Economic Indexes for Areas.

^a^ Adjusted for age, sex, SEIFA, alcohol consumption, dietary pattern, dieting to lose weight, physical activity, and employment. The analysis for former smokers was adjusted for duration of follow-up after smoking cessation; analysis for continuing smokers and never smokers was adjusted for duration of follow-up.

### Smoking Cessation, Weight Change, and Risk of T2DM, CVD, Cancer, COPD, and All-Cause Mortality

During a mean (SD) of 6.0 (3.8) years of follow-up after quitting smoking, there were 1355 CVD, 865 type 2 diabetes, 1387 cancer and 812 COPD incident cases, and 1889 deaths from any cause. Compared with continuing smokers, weight gain following smoking cessation did not significantly increase the risk of CVD, type 2 diabetes, cancer, and COPD (Table 3). However, smoking cessation was associated with significantly lower risk of death regardless of weight change after quitting (Table 4), with the hazard ratios for the risk of death being 0.50 (95% CI, 0.36-0.68) among those who quit who lost weight, 0.79 (95% CI, 0.51-0.98) among those who quit without weight change, 0.33 (95% CI, 0.21-0.51) among those who quit who gained 0.1 to 5.0 kg, 0.24 (95% CI, 0.11-0.53) among those who quit who gained 5.1 to 10 kg, and 0.36 (95% CI, 0.16-0.82) among those who quit who gained more than 10 kg.

**Table 3.** Association Between Weight and BMI Change and the Risk of Cardiovascular Disease, Type 2 Diabetes, and Cancer^a^

Characteristics	CVD		Type 2 diabetes		Cancer	
	HR (95% CI)	*P* value	HR (95% CI)	*P* value	HR (95% CI)	*P* value
Smoking status						
Continuing	1 [Reference]	[Reference]	1 [Reference]	[Reference]	1 [Reference]	[Reference]
Never	0.86 (0.76-0.98)	.02	0.71 (0.55-0.92)	.009	0.90 (0.60-1.34)	.63
Former	0.85 (0.54-1.33)	.48	0.99 (0.78-1.30)	.95	0.95 (0.64-1.41)	.81
Former smokers only						
No weight change	1 [Reference]	[Reference]	1 [Reference]	[Reference]	1 [Reference]	[Reference]
Lost weight	1.23 (0.81-1.89)	.32	1.28 (0.82-2.00)	.27	1.36 (0.88-2.12)	.16
Gained 0.1-5.0 kg	1.31 (0.84-2.03)	.22	0.73 (0.44-1.20)	.22	0.94 (0.58-1.51)	.80
Gained 5.1-10.0 kg	1.56 (0.58-1.68)	.98	0.90 (0.51-1.59)	.73	1.37 (0.81-2.32)	.24
Gained >10 kg	1.49 (0.82-2.73)	.19	1.05 (0.58-1.88)	.87	1.50 (0.85-2.64)	.16
Former smokers vs never smoked						
Nonsmokers	1 [Reference]	[Reference]	1 [Reference]	[Reference]	1 [Reference]	[Reference]
Lost weight	1.14 (0.94-1.41)	.18	1.03 (0.93-2.07)	.32	1.20 (0.96-1.49)	.10
No weight change	0.95 (0.66-1.37)	.80	1.06 (0.53-2.11)	.86	0.88 (0.58-1.33)	.54
Gained 0.1-5.0 kg	1.21 (0.96-1.52)	.10	1.07 (0.67-1.72)	.78	0.82 (0.62-1.09)	.19
Gained 5.1-10.0 kg	1.06 (0.73-1.53)	.76	1.40 (0.77-2.57)	.27	1.20 (0.84-1.72)	.30
Gained >10 kg	1.54 (0.96-2.47)	.07	1.71 (0.90-3.23)	.10	1.31 (0.87-1.99)	.19
Former smokers vs continuing smokers						
Current smokers	1 [Reference]	[Reference]	1 [Reference]	[Reference]	1 [Reference]	[Reference]
Lost weight	0.91 (0.71-1.16)	.44	1.19 (0.75-1.91)	.45	0.84 (0.45-1.55)	.57
No weight change	0.75 (0.51-1.11)	.15	0.75 (0.35-1.59)	.45	0.88 (0.65-3.25)	.36
Gained 0.1-5.0 kg	0.96 (0.75-1.25)	.76	0.72 (0.41-1.27)	.26	0.74 (0.36-1.52)	.42
Gained 5.1-10.0 kg	0.84 (0.56-1.23)	.37	0.93 (0.47-1.82)	.83	0.55 (0.19-1.57)	.27
Gained >10 kg	1.21 (0.74-1.98)	.43	1.08 (0.54-2.18)	.82	0.75 (0.22-2.48)	.64
Former smokers only						
No BMI change	1 [Reference]	[Reference]	1 [Reference]	[Reference]	1 [Reference]	[Reference]
Loss BMI	1.18 (0.72-1.94)	.50	1.18 (0.67-2.11)	.59	1.07 (0.62-1.83)	.80
Gained 0.1-2.0	0.98 (0.59-1.64)	.97	0.82 (0.45-1.51)	.54	1.07 (0.62-1.85)	.81
Gained >2.0	1.02 (0.60-1.75)	.92	0.89 (0.48-1.65)	.73	1.11 (0.63-1.96)	.70
Former smokers vs never smoked						
Non-smokers	1 [Reference]	[Reference]	1 [Reference]	[Reference]	1 [Reference]	[Reference]
Lost BMI	1.05 (0.93-1.19)	.42	1.63 (1.29-2.07)	<.001	1.06 (0.85-1.31)	.60
No BMI change	0.79 (0.57-1.11)	.18	1.38 (0.78-2.41)	.26	0.99 (0.58-1.66)	.96
Gained 0.1-2.0	1.15 (1.01-1.32)	.03	1.14 (0.85-1.53)	.39	1.05 (0.83-1.34)	.66
Gained >2.0	1.21 (1.05-1.39)	.005	1.23 (0.91-1.67)	.17	1.01 (0.85-1.44)	.46
Former smokers vs never smoked						
Current smokers	1 [Reference]	[Reference]	1 [Reference]	[Reference]	1 [Reference]	[Reference]
Lost BMI	1.05 (0.82-1.36)	.66	1.27 (0.94-1.72)	.11	0.98 (0.74-1.29)	.87
No BMI change	0.89 (0.54-1.47)	.66	1.07 (0.59-1.94)	.82	0.91 (0.53-1.58)	.74
Gained 0.1-2.0	0.88 (0.67-1.16)	.38	0.88 (0.63-1.25)	.50	0.97 (0.72-1.31)	.86
Gained >2.0	0.91 (0.67-1.25)	.59	0.96 (0.68-1.36)	.83	1.02 (0.75-1.39)	.90

Abbreviations: BMI, body mass index (calculated as weight in kilograms divided by height in meters squared); CVD, cardiovascular diseases (heart or circulatory diseases); HR, hazard ratio.

^a^ Adjusted for age, sex, SEIFA, alcohol consumption, dietary pattern, dieting to lose weight, physical activity, and employment.

**Table 4.** Association Between Weight and BMI Change and the Risk of Chronic Obstructive Pulmonary Disease and Mortality^a^

Characteristics	COPD		Mortality	
	HR (95% CI)	*P* value	HR (95% CI)	*P* value
Smoking status				
Continuing smokers	1 [Reference]	[Reference]	1 [Reference]	[Reference]
Never smoked	0.18 (0.10-0.33)	<.001	0.36 (0.21-0.59)	<.001
Former smokers	0.84 (0.24-1.24)	.21	0.50 (0.30-0.81)	.005
Former smokers only				
No weight change	1 [Reference]	[Reference]	1 [Reference]	[Reference]
Lost weight	0.95 (0.35-2.61)	.93	0.81 (0.49-1.32)	.41
Gained 0.1-5.0 kg	0.90 (0.30-2.70)	.85	0.55 (0.31-1.00)	.40
Gained 5.1-10.0 kg	0.91 (0.24-3.41)	.89	0.41 (0.17-1.01	.17
Gained >10 kg	0.87 (0.17-4.53)	.87	0.50 (0.35-1.34)	.09
Former smokers vs never smoked				
Nonsmokers	1 [Reference]	[Reference]	1 [Reference]	[Reference]
Lost weight	1.33 (0.74-2.40)	.25	1.35 (0.90-2.04)	.14
No weight change	1.10 (0.50-2.41)	.71	1.85 (0.88-3.89)	.10
Gained 0.1-5.0 kg	1.37 (0.72-2.63)	.33	1.21 (0.69-2.11)	.50
Gained 5.1-10.0 kg	1.49 (0.62-3.50)	.39	1.01 (0.36-2.81)	.98
Gained >10 kg	1.46 (0.61-2.64)	.07	1.41 (0.56-2.99)	.38
Former smokers vs never smoked				
Continuing smokers	1 [Reference]	[Reference]	1 [Reference]	[Reference]
Lost weight	0.54 (0.33-1.01)	.11	0.50 (0.36-0.68)	<.001
No weight change	0.47 (0.23-1.05)	.31	0.79 (0.51-0.98)	.02
0.1-5.0 kg	0.55 (0.31-1.95)	.20	0.33 (0.21-0.51)	<.001
5.1-10.0 kg	0.59 (0.27-1.25)	.17	0.24 (0.11-0.53)	<.001
>10 kg	0.90 (0.41-2.02)	.82	0.36 (0.16-0.82)	.01
Former smokers only				
No BMI change	1 [Reference]	[Reference]	1 [Reference]	[Reference]
Loss BMI	1.49 (0.34-6.53)	.59	0.88 (0.42-1.85)	.75
Gained 0.1-2.0	1.07 (0.23-5.04)	.93	0.61 (0.27-1.38)	.24
Gained >2	1.87 (0.41-8.62)	.42	0.47 (0.19-1.17)	.11
Former smokers vs never smoked				
Lost BMI	1 [Reference]	[Reference]	1 [Reference]	[Reference]
Never smoked	0.55 (0.23-1.70)	.10	1.49 (0.98-2.17)	.39
No BMI change	0.62 (0.32-1.16)	.14	1.06 (0.94-4.54)	.07
Gained 0.1-2.0	0.68 (0.45-1.00)	.06	1.11 (0.62-1.96)	.72
Gained >2	0.95 (0.68-1.33)	.80	1.17 (0.27-1.32)	.21
Former smokers vs continuing smokers				
Continuing smoker	1 [Reference]	[Reference]	1 [Reference]	[Reference]
Lost BMI	0.49 (0.24-1.01)	.06	0.61 (0.45-0.83)	.002
No BMI change	0.49 (0.11-2.17)	.35	0.86 (0.51-0.95)	.005
Gained 0.1-2.0	0.40 (0.17-0.94)	.04	0.32 (0.21-0.50)	<.001
Gained >2	0.42 (0.18-1.00)	.05	0.26 (0.16-0.45)	<.001

Abbreviations: BMI, body mass index (calculated as weight in kilograms divided by height in meters squared); COPD, chronic obstructive pulmonary disease; HR, hazard ratios; SEIFA, Socio-Economic Indexes for Areas.

^a^ Adjusted for age, sex, SEIFA, alcohol consumption, dietary pattern, dieting to lose weight, physical activity, and employment.

When analysis was restricted to those who quit smoking, change in weight after smoking cessation was not significantly associated with the risk of CVD, type 2 diabetes, cancer, and COPD compared with those who quit without weight gain. Compared with those who quit smoking without experiencing weight gain, the hazard ratios for the risk of CVD were 1.23 (95% CI, 0.81 to 1.89) among those who quit who lost weight, 1.31 (95% CI, 0.84 to 2.03) among those who quit who gained 0.1 to 5 kg, 1.56 (95% CI, 0.58 to 1.68) among those who quit who gained 5.1 to 10 kg, and 1.49 (95% CI, 0.82 to 2.73) among those who quit who gained more than 10 kg (Tables 3 and 4). Additionally, change in weight after smoking cessation was not significantly associated with the risk of death (Table 4).

### Smoking Cessation, BMI Change, and Risk of Type 2 Diabetes, CVD, Cancer, COPD, and All-Cause Mortality

Compared with continuing smoking, BMI gain following smoking cessation was not associated with a significant increase in the risk of CVD, type 2 diabetes, cancer, and COPD (Tables 3 and 4). In contrast, those who quit smoking had a significantly lower risk of death regardless of BMI change compared with those who continued smoking, with the HRs for the risk of death being 0.61 (95% CI, 0.45-0.83) among those who quit who lost BMI, 0.86 (95% CI, 0.51-1.44) among those who quit without change in BMI, 0.32 (95% CI, 0.21-0.50) among those who quit who gained BMI up to 2 after quitting, and 0.26 (95% CI, 0.16-0.45) among those who quit who gained more than 2 (Tables 3 and 4). In the analyses restricted to participants who quit smoking, BMI change after smoking cessation was not significantly associated with the risk of CVD, type 2 diabetes, cancer, and COPD (Tables 3 and 4). Compared with those who quit without BMI gain, the HRs for the risk of CVD were 1.18 (95% CI, 0.72-1.94) among those who quit whose BMI decreased, 0.98 (95% CI, 0.59-1.64) among those who quit who gained BMI between 0.1 and 2, and 1.02 (95% CI, 0.60-1.75) among those who quit who gained more than 2.

In the sensitivity analysis, the findings of lack of an association between weight and BMI gain following smoking cessation and risk of CVD, type 2 diabetes, cancer, COPD, and all-cause mortality did not change when those who quit for less than 2 years were excluded from the analyses (eTables 1-3 in the Supplement). The findings did not substantially change when longitudinal weights were applied to account for the loss of sample members from a longitudinal survey (eTables 4-6 in the Supplement). Results of the analyses using the imputed data were largely consistent with the results using only complete cases (eTables 7-9 in the Supplement).

## Discussion

Based on a nationally representative longitudinal study of 16 663 adults, we found that smoking cessation is significantly associated with weight and BMI gain. However, the benefits of smoking cessation outweighed the risks in terms of reducing mortality in the general population because the risks of the major chronic diseases did not increase regardless of the amount of weight and BMI change after quitting.

Our results complement findings from previous studies that reported significant weight^5,6,28^ and BMI gains^6,28,29^ among adults who quit smoking compared with continuing smokers. The excess weight and BMI gain among those who quit, compared with continuing smokers, ranges from 1 kg to 10 kg^4,17,28,30^ and 0.1 to 2.0,^2,6,31^ respectively. Overall, studies that have a longer follow-up time since quitting tend to report more weight gain among those who quit,^5,17,28^ as weight gain increases with increasing duration of quitting smoking before gradually decreasing.^5,28^ Furthermore, the rate of increase in weight or BMI after quitting smoking may vary depending on whether those who quit had received pharmacotherapy for smoking cessation^17^ or received weight management programs, including physical activity and healthy diet.^8^

However, the added value of our results is the finding that adults who quit smoking have significantly lower risk of death than continuing smokers regardless of the amount of weight and BMI change after quitting. Most importantly, there was a significant reduction in the risk of death among former smokers independent of whether or not they gained weight and BMI. In the present study, the proportion of those who quit who gained 10 kg or more (30.5%) and 2 or more BMI units (32.1%) were nearly twice of what was reported in previous studies,^4,6,10,32^ which might have reduced the protective benefits of quitting smoking in terms of reducing the risk of chronic diseases.

Our findings suggest that the long-term benefits of smoking cessation considerably outweigh the risks associated with postcessation weight or BMI gain. This is consistent with previous studies.^1,5,12^ A prospective study of 1.3 million UK women also showed that former smokers who permanently stopped smoking have excess (compared with never smokers) risk of all-cause mortality and mortality from lung cancer; however, the excess risk among former smokers was only 3% to 10% of the excess mortality compared with continuing smokers.^1^

Nonetheless, numerous studies have produced findings that do not agree with our results. This could be due to differences in the characteristics of studied populations, the operative definition and selection of risk factors, the range and operational definition of included chronic diseases, and the length of follow-up. For example, Hu et al^5^ found that weight gain due to smoking cessation is associated with a 22% increased risk of type 2 diabetes, but a significantly lower risk of death, 2 to 6 years after quitting.^5^ The authors noted that the health risks were directly proportional to the amount of weight gained, but decreased as time passed since smoking cessation.^5^ However, this study was based on 3 large cohorts of US health professionals rather than the general population. Similarly, a 2015 meta-analysis of prospective studies^20^ also found that compared with people who never smoked, former smokers had a 14% higher risk of type 2 diabetes, but that that risk decreased substantially as the time since quitting increased. In another study, using data from the Framingham Offspring Study, people who quit smoking who gained 5 kg or more had no reduction in CVD risk compared with continuing smokers, while among participants who lost weight and those who gained 0 to 5 kg, the risk of CVD was only significantly lower for long-term (>4 years) former smokers.^9^

From clinical and public health perspectives, our findings suggest that public health interventions to promote smoking cessation in the population should point out that the health benefits of quitting far exceed any adverse health effects of weight gain. In light of the considerable variability in the amount of weight gain according to duration of smoking cessation, clinical guidelines for smoking cessation may need to include recommendations regarding the timing and duration of postcessation weight management interventions.

### Limitations

This study has several limitations that should be noted. Onset of CVD, type 2 diabetes, cancer, and COPD was defined based on self-reported physician’s diagnosis, which could lead to underreporting. Nevertheless, the validity of self-reported health risk factors and chronic diseases in epidemiological studies has been demonstrated.^33,34^ Weight and BMI were based on self-report and there is a possibility that they might have been underreported. However, the distribution of BMI scores in the HILDA survey participants is comparable with the BMI data for the Australian population. Definition of smoking cessation relied on self-report and it is possible that there has been misclassification of quitting smoking. Furthermore, the duration of follow-up may not be sufficiently long to observe the long-term development of chronic diseases, hence possibly leading to the underestimation of the associations. Further studies with longer follow-up time are required to confirm our findings. Residual confounding is also possible due to unmeasured variables such as environmental risk factors and interventions for smoking cessation and weight control.

## Conclusions

In this cohort of a community-based, nationally representative sample, smoking cessation was significantly associated with weight and BMI gains compared with continuing smoking. However, adults who quit had a significantly lower risk of death than those who continued to smoke regardless of the amount of weight and BMI gain. Neither weight nor BMI gain that occurred following smoking cessation was associated with an increased risk of CVD, type 2 diabetes, cancer, and COPD. The findings support that the benefits of smoking cessation outweigh the risks in terms of reducing mortality in the general population without increasing the risks of the major chronic diseases.

## Notes

Supplement. **eTable 1.** Analyses Excluding Those Less 2 Years Since Quitting: Association Between Smoking Cessation and Weight and BMI Change **eTable 2.** Analyses Excluding Those Less 2 Years Since Quitting: Association Between Weight and BMI Change and the Risk of Cardiovascular Disease, Type 2 Diabetes, and Cancer **eTable 3.** Analyses Excluding Those Less 2 Years Since Quitting: Association Between Weight and BMI Change and the Risk of Chronic Obstructive Pulmonary Disease and Mortality **eTable 4.** Analyses Based on Longitudinal Weights: Association Between Smoking Cessation and Weight and BMI Change **eTable 5.** Analyses Based on Longitudinal Weights: Association Between Weight and BMI Change and the Risk of Cardiovascular Disease, Type 2 Diabetes, and Cancer **eTable 6.** Analyses Based on Longitudinal Weights: Association Between Weight and BMI Change and the Risk of Chronic Obstructive Pulmonary Disease and Mortality **eTable 7.** Analyses Based on Multiple-Imputed Values for Missing Covariates: Association Between Smoking Cessation and Weight and BMI Change **eTable 8.** Analyses Based on Multiple-Imputed Values for Missing Covariates: Association Between Weight and BMI Change and the Risk of Cardiovascular Disease, Type 2 Diabetes, and Cancer **eTable 9.** Analyses Based on Multiple-Imputed Values for Missing Covariates: Association Between Weight and BMI Change and the Risk of Chronic Obstructive Pulmonary Disease and Mortality
