# Supplementary material for: Weight Gain After Smoking Cessation and Risk of Major Chronic Diseases and Mortality
Source: JAMA Netw Open. 2021 Apr 27;4(4):e217044. doi: 10.1001/jamanetworkopen.2021.7044 (PMC8080225; doi:10.1001/jamanetworkopen.2021.7044)
Supplement: Supplement. — eTable 1. Analyses Excluding Those Less 2 Years Since Quitting: Association Between Smoking Cessation and Weight and BMI Change eTable 2. Analyses Excluding Those Less 2 Years Since Quitting: Association Between Weight and BMI Change and the Risk of Cardiovascular Disease, Type 2 Diabetes, and Cancer eTable 3. Analyses Excluding Those Less 2 Years Since Quitting: Association Between Weight and BMI Change and the Risk of Chronic Obstructive Pulmonary Disease and Mortality eTable 4. Analyses Based on Longitudinal Weights: Association Between Smoking Cessation and Weight and BMI Change eTable 5. Analyses Based on Longitudinal Weights: Association Between Weight and BMI Change and the Risk of Cardiovascular Disease, Type 2 Diabetes, and Cancer eTable 6. Analyses Based on Longitudinal Weights: Association Between Weight and BMI Change and the Risk of Chronic Obstructive Pulmonary Disease and Mortality eTable 7. Analyses Based on Multiple-Imputed Values for Missing Covariates: Association Between Smoking Cessation and Weight and BMI Change eTable 8. Analyses Based on Multiple-Imputed Values for Missing Covariates: Association Between Weight and BMI Change and the Risk of Cardiovascular Disease, Type 2 Diabetes, and Cancer eTable 9. Analyses Based on Multiple-Imputed Values for Missing Covariates: Association Between Weight and BMI Change and the Risk of Chronic Obstructive Pulmonary Disease and Mortality [file jamanetwopen-e217044-s001.pdf]

Supplemental Online Content

Sahle BW, Chen W, Rawal LB, Renzaho AMN. Weight gain after smoking cessation and risk of major chronic diseases and mortality. *JAMA Netw Open.* 2021;4(4):e217044. doi:10.1001/jamanetworkopen.2021.7044

eTable 1. Analyses Excluding Those Less 2 Years Since Quitting: Association Between Smoking Cessation and Weight and BMI Change

eTable 2. Analyses Excluding Those Less 2 Years Since Quitting: Association Between Weight and BMI Change and the Risk of Cardiovascular Disease, Type 2 Diabetes, and Cancer

eTable 3. Analyses Excluding Those Less 2 Years Since Quitting: Association Between Weight and BMI Change and the Risk of Chronic Obstructive Pulmonary Disease and Mortality

eTable 4. Analyses Based on Longitudinal Weights: Association Between Smoking Cessation and Weight and BMI Change

eTable 5. Analyses Based on Longitudinal Weights: Association Between Weight and BMI Change and the Risk of Cardiovascular Disease, Type 2 Diabetes, and Cancer

eTable 6. Analyses Based on Longitudinal Weights: Association Between Weight and BMI Change and the Risk of Chronic Obstructive Pulmonary Disease and Mortality

eTable 7. Analyses Based on Multiple-Imputed Values for Missing Covariates: Association Between Smoking Cessation and Weight and BMI Change

eTable 8. Analyses Based on Multiple-Imputed Values for Missing Covariates: Association Between Weight and BMI Change and the Risk of Cardiovascular Disease, Type 2 Diabetes, and Cancer

eTable 9. Analyses Based on Multiple-Imputed Values for Missing Covariates: Association Between Weight and BMI Change and the Risk of Chronic Obstructive Pulmonary Disease and Mortality

This supplemental material has been provided by the authors to give readers additional information about their work.

© 2021 Sahle BW et al. *JAMA Network Open.*

eTable 1. Analyses excluding those less two years since quitting: association between smoking cessation and weight and BMI change

	Weight change, kg		BMI change, kg/m2	
	β (95% CI)	*P*	β (95% CI)	*P*
Smoking status				
Continuing smokers	Ref		Ref	
Never-smoked	2.14 (-0.32, 4.61)	0.088	0.43 (-0.45, 1.13)	0.139
Ex-smokers	3.98 (1.62, 6.34)	0.001	1.11 (0.271, 1.96)	0.010
Quadratic interaction term (Never-smoked*duration)	-0.21 (-0.51, 08)	0.154	-0.06 (-0.16, 0.04)	0.237
Quadratic interaction term (Ex-smokers *duration)	-0.38 (-0.66, -0.09)	0.010	-0.11 (-0.21, -0.01)	0.026

Adjusted for age, sex, SEIFA, alcohol consumption, dietary pattern, dieting to lose weight, physical activity, employment. Duration of follow-up after smoking cessation for quitters, and duration of follow-up for continuing smokers and never-smokers.

© 2021 Sahle BW et al. *JAMA Network Open.*

eTable 2. Analyses excluding those less two years since quitting: association between weight and BMI change and the risk of cardiovascular disease, type 2 diabetes, and cancer

Characteristics	CVD		T2DM		Cancer	
	HR (95% CI)	P	HR (95% CI)	P	HR (95% CI)	P
Smoking status						
Continuing smokers	Ref		Ref		Ref	
Never-smoked	0.79 (0.67, 0.93)	0.006	0.85 (0.65, 0.89)	0.018	0.95 (0.63, 1.41)	0.802
Ex-smokers	0.92 (0.78, 1.10)	0.404	1.08 (0.84, 1.38)	0.952	0.86 (0.56, 1.33)	0.520
Ex-smokers only						
Lost weight	1.22 (0.81, 1.86)	0.330	1.27 (0.77, 2.09)	0.342	0.72 (0.34, 1.52)	0.390
No weight change	Ref		Ref		Ref	
0.1-5.0 kg	1.32 (0.86, 2.03)	0.200	0.84 (0.48, 1.47)	0.555	0.70 (0.31, 1.59)	0.395
5.1-10.0 kg	1.11 (0.66, 1.85)	0.697	0.78 (0.40, 1.48)	0.445	1.07 (0.80, 1.75)	0.309
>10 kg	1.57 (0.86, 2.86)	0.133	1.21 (0.62, 2.33)	0.573	1.20 (0.32, 2.64)	0.968
Ex-smokers vs never-smoked						
Never-smoked	Ref		Ref		Ref	
Lost weight	1.08 (0.87, 1.34)	0.450	1.79 (1.39, 2.31)	<0.001	0.90 (0.58, 1.46)	0.727
No weight change	0.87 (0.59, 1.30)	0.515	1.38 (0.86, 2.22)	0.175	1.27 (0.67, 2.44)	0.457
0.1-5.0 kg	1.15 (0.90, 1.46)	0.254	1.14 (0.80, 1.62)	0.457	0.89 (0.50, 1.58)	0.702
5.1-10.0 kg	1.04 (0.59, 1.26)	0.472	1.04 (0.64, 1.69)	0.855	1.12 (0.25, 1.90)	0.483
>10 kg	1.31 (0.81, 1.11)	0.263	1.50 (0.91, 2.48)	0.111	1.30 (0.48, 3.56)	0.597
Continuing smokers	1.21 (98, 1.49)	0.075				
Ex-smokers vs smokers						
Continuing smokers	Ref		Ref		Ref	
Lost weight	0.92 (0.56, 1.49)	0.741	1.36 (0.81, 2.31)	0.343	0.84 (0.45, 1.55)	0.581
No weight change	0.82 (0.38, 1.76)	0.613	1.00 (0.43, 2.35)	0.431	0.78 (0.66, 3.35)	0.325
0.1-5.0 kg	0.92 (0.53, 1.58)	0.771	1.24 (0.69, 2.23)	0.840	0.77 (0.38, 1.52)	0.477
5.1-10.0 kg	0.86 (0.35, 1.35)	0.285	1.23 (0.56, 1.61)	0.860	0.54 (0.19, 1.56)	0.258
>10 kg	1.26 (0.54, 2.90)	0.585	1.52 (0.68, 3.41)	0.143	0.74 (0.22, 2.45)	0.623
Ex-smokers vs never-smoked						
Never-smoked	Ref		Ref		Ref	
Lost BMI	1.18 (0.96, 1.45)	0.106	1.64 (1.27, 2.11)	0.000	0.89 (0.54, 1.50)	0.681
No BMI change	1.00 (0.62, 1.60)	0.998	1.11 (0.57, 2.16)	0.758	1.01 (0.90, 1.85)	0.082
0.1-2.0 kg m^{-2}	0.99 (0.78, 1.24)	0.923	1.11 (0.80, 1.52)	0.519	1.15 (0.42, 1.38)	0.374
>2 kg m^{-2}	1.02 (0.78, 1.35)	0.849	1.20 (0.86, 1.68)	0.283	1.01 (0.36, 1.84)	0.390
Ex-smokers vs smokers						
Continuing smokers	Ref		Ref		Ref	
Lost BMI	0.97 (0.75, 1.27)	0.872	1.26 (0.91, 1.75)	0.166	0.88 (0.57, 1.57)	0.861
No BMI change	0.80 (0.48, 1.35)	0.416	0.86 (0.42, 1.73)	0.663	1.31 (0.60, 1.85)	0.440
0.1-2.0 kg m^{-2}	0.81 (0.60, 1.07)	0.150	0.85 (0.58, 1.25)	0.422	1.02 (0.48, 1.50)	0.579
> 2 kg m^{-2}	0.79 (0.58, 1.09)	0.165	0.92 (0.62, 1.36)	0.697	1.21 (0.35, 1.42)	0.349

HR (95% CI), hazard ratios (95% confidence interval); BMI, body mass index (Kg/m2); CVD, cardiovascular diseases (heart or circulatory diseases); T2DM, type 2 diabetes. Adjusted for age, sex, SEIFA, alcohol consumption, dietary pattern, dieting to lose weight, physical activity, employment. The association between smoking status and risk of CVD, T2DM, and

© 2021 Sahle BW et al. *JAMA Network Open.*

cancer was not modified by weight change. The association between weight gain after quitting and risk of CVD, T2DM, and cancer was not modified by duration of follow-up or duration since smoking cessation.

© 2021 Sahle BW et al. *JAMA Network Open.*

eTable 3: Analyses excluding those less two years since quitting: association between weight and BMI change and the risk of chronic obstructive pulmonary disease, and mortality

Characteristics	COPD		Mortality	
	HR (95% CI)	P	HR (95% CI)	P
Smoking status				
Continuing smokers	Ref		Ref	
Never-smoked	0.24 (0.21, 0.63)	0.001	0.37 (0.28, 0.49)	<0.001
Ex-smokers	0.89 (0.37, 1.72)	0.312	0.45 (0.35, 0.59)	<0.001
Ex-smokers only				
Lost weight	0.84 (0.48, 1.41)	0.416	0.81 (0.49, 1.32)	0.410
No weight change	Ref		Ref	
0.1-5.0 kg	0.85 (0.43, 1.97)	0.623	0.55 (0.31, 1.00)	0.404
5.1-10.0 kg	0.91 (0.24, 3.41)	0.217	0.41 (0.17, 1.01	0.170
>10 kg	0.97 (0.35, 2.43)	0.243	0.50 (0.35, 1.34)	0.090
Ex-smokers vs Never-smoked				
Never-smoked	Ref	Ref	Ref	Ref
Lost weight	1.11 (0.65, 1.78)	0.136	1.31 (0.99, 1.73)	0.056
No weight change	1.03 (0.48, 2.12)	0.706	1.57 (0.95, 2.59)	0.078
0.1-5.0 kg	1.21 (0.64, 2.41)	0.331	0.81 (0.51, 1.26)	0.347
5.1-10.0 kg	1.17 (0.53, 1.94)	0.390	0.71 (0.33, 1.52)	0.382
>10 kg	1.34 (0.42, 2.47)	0.218	0.53 (0.16, 1.67)	0.297
Ex-smokers vs smokers				
Continuing smokers	Ref		Ref	
Lost weight	0.54 (0.24, 1.27)	0.114	0.47 (0.36, 0.82)	0.008
No weight change	0.57 (0.28, 1.63)	0.216	0.60 (0.51, 0.98)	0.024
0.1-5.0 kg	0.46 (0.18, 1.91)	0.104	0.33 (0.21, 0.69)	0.003
5.1-10.0 kg	0.63 (0.37, 1.41)	0.168	0.37 (0.11, 0.86)	0.017
>10 kg	1.02 (0.41, 2.04)	0.414	0.45 (0.16, 0.87)	0.010
Ex-smokers vs never-smoked				
Never-smoked	Ref		Ref	
Lost BMI	0.61 (0.28, 1.72)	0.201	1.37 (0.98, 1.75)	0.218
No BMI change	0.71 (0.43, 1.99)	0.142	1.08 (0.49, 3.19)	0.120
0.1-2.0 kg m^{-2}	0.68 (0.38, 1.89)	0.112	0.73 (0.34, 1.97)	0.136
>2 kg m^{-2}	0.88 (0.47, 2.01)	0.206	2.16 (0.27, 3.45)	0.206
Ex-smokers vs smokers				
Continuing smokers	Ref		Ref	
Lost BMI	0.53 (0.18, 1.73)	0.345	0.52 (0.38, 0.71)	<0.001
No BMI change	0.67 (0.27, 1.78)	0.213	0.65 (0.34, 0.93)	<0.001
0.1-2.0 kg m^{-2}	0.72 (0.18, 1.97)	0.501	0.74 (0.18, 0.95)	<0.001
>2 kg m^{-2}	0.53 (0.17, 1.88)	0.218	0.48 (0.26, 0.85)	<0.001

HR (95% CI), hazard ratios (95% confidence interval); BMI, body mass index (Kg/m2); COPD, chronic obstructive pulmonary disease. Adjusted for age, sex, SEIFA, alcohol consumption, dietary pattern, dieting to lose weight, physical activity, employment. The association between smoking status and risk of COPD and mortality was not modified by weight change. The association between weight gain after quitting and risk of COPD and mortality was not modified by duration of follow-up or duration since smoking cessation.

© 2021 Sahle BW et al. *JAMA Network Open.*

eTable 4. Analyses based on longitudinal weights: association between smoking cessation and weight and BMI change

	Weight change, kg		BMI change, kg/m2	
	β (95% CI)	*P*	β (95% CI)	*P*
Smoking status				
Continuing smokers	Ref		Ref	
Never-smoked	2.01 (-0.43, 3.97)	0.108	0.38 (-0.17, 1.65)	0.211
Ex-smokers	3.45 (1.47, 6.12)	0.013	1.08 (0.27, 2.01)	0.009
Quadratic interaction term (Never-smoked*duration)	-0.11 (-0.35, 07)	0.213	-0.03 (-0.13, 0.10)	0.213
Quadratic interaction term (Ex-smoking *duration)	-0.29 (-0.48, -0.06)	0.007	-0.10 (-0.17, -0.08)	0.011

Adjusted for age, sex, SEIFA, alcohol consumption, dietary pattern, dieting to lose weight, physical activity, employment. Duration of follow-up after smoking cessation for quitters, and duration of follow-up for continuing smokers and never-smokers.

© 2021 Sahle BW et al. *JAMA Network Open.*

eTable 5. Analyses based on longitudinal weights: association between weight and BMI change and the risk of cardiovascular disease, type 2 diabetes, and cancer

Characteristics	CVD		T2DM		Cancer	
	HR (95% CI)	P	HR (95% CI)	P	HR (95% CI)	P
Smoking status						
Continuing smokers	Ref		Ref		Ref	
Never-smoked	0.65 (0.36, 0.89)	0.010	0.78 (0.48, 0.92)	0.009	0.88 (0.56, 1.76)	0.415
Ex-smokers	0.78 (0.46, 1.28)	0.297	1.18 (0.56, 2.00)	0.413	0.91 (0.56, 2.33)	0.702
Ex-smokers only						
Lost weight	1.18 (0.73, 1.81)	0.315	1.31 (0.45, 2.79)	0.203	1.01 (0.80, 2.93)	0.197
No weight change	Ref		Ref		Ref	
0.1-5.0 kg	1.13 (0.54, 1.98)	0.204	0.73 (0.34, 1.84)	0.387	1.33 (0.73, 3.01)	0.255
5.1-10.0 kg	1.41 (0.73, 2.14)	0.544	0.83 (0.50, 1.51)	0.445	1.50 (0.59, 2.75)	0.657
>10 kg	1.44 (0.72, 3.51)	0.301	1.18 (0.49, 2.00)	0.340	1.39 (0.80, 3.12)	0.723
Ex-smokers vs never-smoked						
Never-smoked	Ref		Ref		Ref	
Lost weight	0.98 (0.67, 1.54)	0.320	1.23 (0.97, 2.31)	0.060	0.90 (0.58, 1.46)	0.413
No weight change	0.88 (0.45, 1.83)	0.413	1.07 (0.79, 2.22)	0.175	1.27 (0.67, 2.54)	0.294
0.1-5.0 kg	1.17 (0.77, 1.81)	0.191	1.11 (0.83, 1.62)	0.457	0.89 (0.50, 1.78)	0.503
5.1-10.0 kg	1.08 (0.43, 1.74)	0.501	1.20 (0.49, 1.69)	0.233	1.12 (0.93, 2.89)	0.414
>10 kg	1.28 (0.68, 2.17)	0.161	1.34 (0.57, 2.03)	0.111	1.51 (0.65, 3.71)	0.679
Ex-smokers vs smokers						
Continuing smokers	Ref		Ref		Ref	
Lost weight	0.68 (0.38, 1.57)	0.232	1.14 (0.54, 1.88)	0.255	0.86 (0.33, 2.11)	0.391
No weight change	0.87 (0.56, 2.32)	0.155	1.03 (0.28, 2.12)	0.302	0.79 (0.46, 3.09)	0.145
0.1-5.0 kg	0.92 (0.47, 1.76)	0.343	1.09 (0.43, 1.91)	0.614	0.89 (0.51, 2.88)	0.215
5.1-10.0 kg	0.81 (0.33, 1.91)	0.108	1.17 (0.51, 1.70)	0.532	0.81 (0.39, 1.73)	0.391
>10 kg	1.21 (0.56, 2.13)	0.579	1.21 (0.65, 2.81)	0.251	1.09 (0.43, 3.42)	0.701
Ex-smokers vs never-smoked						
Never-smoked	Ref		Ref		Ref	
Lost BMI	1.08 (0.67, 1.86)	0.215	1.24 (1.27, 2.11)	0.013	0.93 (0.52, 2.10)	0.232
No BMI change	0.89 (0.54, 1.66)	0.308	1.17 (0.63, 2.16)	0.457	1.02 (0.43, 1.99)	0.324
0.1-2.0 kg m^{-2}	1.01 (0.47, 1.84)	0.249	1.09 (0.50, 1.52)	0.510	1.15 (0.65, 2.16)	0.477
>2 kg m^{-2}	1.09 (0.73, 2.12)	0.461	1.23 (0.71, 1.68)	0.184	1.23 (0.48, 1.87)	0.280
Ex-smokers vs smokers						
Continuing smokers	Ref		Ref		Ref	
Lost BMI	1.00 (0.81, 2.17)	0.482	0.99 (0.73, 1.84)	0.200	0.83 (0.46, 1.88)	0.445
No BMI change	0.89 (0.35, 1.81)	0.413	0.88 (0.35, 2.00)	0.421	0.99 (0.39, 1.74)	0.246
0.1-2.0 kg m^{-2}	0.91 (0.57, 3.01)	0.643	0.73 (0.41, 1.66)	0.310	1.04 (0.52, 2.00)	0.310
>2 kg m^{-2}	0.83 (0.53, 1.67)	0.256	1.00 (0.50, 1.78)	0.521	1.09 (0.27, 1.81)	0.215

HR (95% CI), hazard ratios (95% confidence interval); BMI, body mass index (Kg/m2); CVD, cardiovascular diseases (heart or circulatory diseases); T2DM, type 2 diabetes. Adjusted for age, sex, SEIFA, alcohol consumption, dietary pattern, dieting to lose weight, physical activity, employment. The association between smoking status and risk of CVD, T2DM, and cancer was not modified by weight change. The association between weight gain after quitting and risk of CVD, T2DM, and cancer was not modified by duration of follow-up or duration since smoking cessation.

© 2021 Sahle BW et al. *JAMA Network Open.*

eTable 6. Analyses based on longitudinal weights: association between weight and BMI change and the risk of chronic obstructive pulmonary disease, and mortality

Characteristics	COPD		Mortality	
	HR (95% CI)	P	HR (95% CI)	P
Smoking status				
Continuing smokers	Ref		Ref	
Never-smoked	0.40 (0.17, 0.86)	0.001	0.51 (0.28, 0.83)	0.000
Ex-smokers	0.69 (0.21, 2.31)	0.280	0.67 (0.33, 0.90)	0.003
Ex-smokers vs never-smoked				
Never-smoked	Ref	Ref	Ref	Ref
Lost weight	1.04 (0.37, 1.81)	0.207	1.09 (1.00, 2.45)	0.189
No weight change	0.98 (0.31, 1.70)	0.192	1.03 (0.97, 2.39)	0.214
0.1-5.0 kg	1.15 (0.51, 2.67)	0.237	0.92 (0.45, 1.67)	0.312
5.1-10.0 kg	1.22 (0.67, 3.72)	0.541	1.11 (0.63, 2.75)	0.382
>10 kg	1.64 (0.59, 2.12)	0.318	1.43 (0.49, 3.94)	0.540
Ex-smokers vs smokers				
Continuing smokers	Ref		Ref	
Lost weight	0.67 (0.43, 1.85)	0.214	0.59 (0.45, 0.91)	0.012
No weight change	0.59 (0.27, 1.45)	0.199	0.64 (0.36, 0.92)	0.011
0.1-5.0 kg	0.44 (0.21, 1.76)	0.218	0.55 (0.18, 0.80)	0.008
5.1-10.0 kg	0.75 (0.39, 1.83)	0.345	0.67 (0.35, 0.89)	0.015
>10 kg	1.10 (0.36, 2.58)	0.527	0.71 (0.28, 0.94)	0.022
Ex-smokers vs never-smoked				
Never-smoked	Ref		Ref	
Lost BMI	0.86 (0.41, 2.46)	0.305	1.21 (0.79, 3.27)	0.309
No BMI change	0.91 (0.51, 2.35)	0.211	1.13 (0.38, 2.93)	0.255
0.1-2.0 kg m^{-2}	1.01 (0.60, 1.94)	0.300	0.89 (0.38, 1.87)	0.145
>2 kg m^{-2}	0.96 (0.53, 2.73)	0.542	1.28 (0.64, 2.78)	0.313
Ex-smokers vs smokers				
Continuing smokers	Ref		Ref	
Lost BMI	0.71 (0.34, 2.90)	0.658	0.61 (0.25, 0.95)	<0.000
No BMI change	0.59 (0.17, 1.69)	0.340	0.68 (0.34, 0.94)	<0.000
0.1-2.0 kg m^{-2}	0.82 (0.45, 3.34)	0.621	0.76 (0.18, 0.89)	< 0.000
>2 kg m^{-2}	0.53 (0.17, 1.88)	0.218	0.48 (0.26, 0.85)	< 0.000

HR (95% CI), hazard ratios (95% confidence interval); BMI, body mass index (Kg/m2); COPD, chronic obstructive pulmonary disease. Adjusted for age, sex, SEIFA, alcohol consumption, dietary pattern, dieting to lose weight, physical activity, employment. The association between smoking status and risk of COPD and mortality was not modified by weight change. The association between weight gain after quitting and risk of COPD and mortality was not modified by duration of follow-up or duration since smoking cessation.

© 2021 Sahle BW et al. *JAMA Network Open.*

eTable 7. Analyses based on multiple-imputed values for missing covariates: association between smoking cessation and weight and BMI change

	Weight change, kg		BMI change, kg/m2	
	β (95% CI)	*P*	β (95% CI)	*P*
Smoking status				
Continuing smokers	Ref		Ref	
Never-smoked	1.93 (-0.55, 3.33)	0.110	0.40 (-0.20, 1.44)	0.129
Ex-smokers	3.56 (1.76, 6.49)	0.006	0.99 (0.29, 1.95)	0.007
Quadratic interaction term (Never-smoked*duration)	-0.09 (-0.39, 08)	0.113	-0.05 (-0.17, 0.19)	0.105
Quadratic interaction term (Ex-smoking *duration)	-0.19 (-0.41, -0.09)	0.010	-0.11 (-0.21, -0.06)	0.009

Adjusted for age, sex, SEIFA, alcohol consumption, dietary pattern, dieting to lose weight, physical activity, employment. Duration of follow-up after smoking cessation for quitters, and duration of follow-up for continuing smokers and never-smoked

© 2021 Sahle BW et al. *JAMA Network Open.*

eTable 8. Analyses based on multiple-imputed values for missing covariates: association between weight and BMI change and the risk of cardiovascular disease, type 2 diabetes, and cancer

Characteristics	CVD		T2DM		Cancer	
	HR (95% CI)	P	HR (95% CI)	P	HR (95% CI)	P
Smoking status						
Continuing smokers	Ref		Ref		Ref	
Never-smoked	0.73 (0.32, 0.94)	0.019	0.74 (0.37, 0.89)	0.000	0.79 (0.46, 1.93)	0.318
Ex-smokers	0.88 (0.44, 2.35)	0.300	1.22 (0.61, 2.46)	0.318	0.95 (0.37, 2.86)	0.546
Ex-smokers vs never-smoked						
Never-smoked	Ref		Ref		Ref	
Lost weight	0.96 (0.49, 2.45)	0.198	1.05 (0.68, 3.14)	0.232	0.92 (0.45, 1.78)	0.314
No weight change	0.97 (0.44, 3.11)	0.547	1.03 (0.55, 2.13)	0.109	1.18 (0.55, 1.97)	0.199
0.1-5.0 kg	1.07 (0.63, 2.20)	0.158	1.09 (0.49, 2.13)	0.371	0.77 (0.29, 2.57)	0.305
5.1-10.0 kg	1.23 (0.76, 3.93)	0.634	1.21 (0.54, 2.40)	0.365	1.11 (0.88, 1.97)	0.341
>10 kg	1.17 (0.40, 3.54)	0.240	1.33 (0.65, 3.07)	0.272	1.33 (0.70, 3.88)	0.470
Ex-smokers vs smokers						
Continuing smokers	Ref		Ref		Ref	
Lost weight	0.73 (0.41, 1.93)	0.187	1.07 (0.64, 1.92)	0.144	0.88 (0.43, 2.71)	0.307
No weight change	0.68 (0.27, 1.60)	0.215	1.00 (0.24, 2.48)	0.215	0.66 (0.36, 2.49)	0.200
0.1-5.0 kg	0.92 (0.44, 2.44)	0.346	1.13 (0.55, 2.58)	0.245	0.90 (0.51, 2.98)	0.311
5.1-10.0 kg	0.89 (0.34, 2.17)	0.260	1.04 (0.30, 1.79)	0.413	0.89 (0.38, 2.31)	0.418
>10 kg	1.09 (0.42, 2.00)	0.416	1.26 (0.51, 3.71)	0.309	1.05 (0.56, 2.97)	0.605
Ex-smokers vs never-smoked						
Never-smoked	Ref		Ref		Ref	
Lost BMI	1.01 (0.67, 1.87)	0.132	1.08 (0.97, 3.02)	0.061	1.00 (0.43, 1.99)	0.200
No BMI change	0.91 (0.46, 1.67)	0.218	0.98 (0.30, 2.09)	0.306	1.08 (0.44, 2.15)	0.118
0.1-2.0 kg m^{-2}	1.03 (0.49, 2.53)	0.403	1.07 (0.18, 1.78)	0.379	1.11 (0.77, 2.88)	0.215
>2 kg m^{-2}	1.32 (0.41, 3.09)	0.355	1.18 (0.65, 1.79)	0.133	1.25 (0.48, 2.00)	0.391
Ex-smokers vs smokers						
Continuing smokers	Ref		Ref		Ref	
Lost BMI	0.98 (0.43, 2.98)	0.415	1.03 (0.43, 3.01)	0.145	1.00 (0.43, 2.33)	0.333
No BMI change	0.72 (0.43, 1.79)	0.267	0.90 (0.55, 2.65)	0.312	0.90 (0.70, 1.89)	0.266
0.1-2.0 kg m^{-2}	0.87 (0.54, 2.99)	0.418	1.06 (0.80, 3.19)	0.289	1.07 (0.40, 2.67)	0.481
>2 kg m^{-2}	1.07 (0.67, 2.59)	0.534	1.13 (0.76, 2.81)	0.355	1.06 (0.39, 2.91)	0.198

HR (95% CI), hazard ratios (95% confidence interval); BMI, body mass index (Kg/m2); CVD, cardiovascular diseases (heart or circulatory diseases); T2DM, type 2 diabetes. Adjusted for age, sex, SEIFA, alcohol consumption, dietary pattern, dieting to lose weight, physical activity, employment. The association between smoking status and risk of CVD, T2DM, and cancer was not modified by weight change. The association between weight gain after quitting and risk of CVD, T2DM, and cancer was not modified by duration of follow-up or duration since smoking cessation.

© 2021 Sahle BW et al. *JAMA Network Open.*

eTable 9. Analyses based on multiple-imputed values for missing covariates: association between weight and BMI change and the risk of chronic obstructive pulmonary disease, and mortality

Characteristics	COPD		Mortality	
	HR (95% CI)	P	HR (95% CI)	P
Smoking status				
Continuing smokers	Ref		Ref	
Never-smoked	0.67 (0.27, 0.90)	0.010	0.50 (0.30, 0.93)	0.000
Ex-smokers	0.89 (0.43, 2.60)	0.200	0.71 (0.35, 0.89)	0.008
Ex-smokers vs never-smoked				
Never-smoked	Ref	Ref	Ref	Ref
Lost weight	0.99 (0.31, 1.93)	0.188	1.05 (0.63, 2.87)	0.234
No weight change	1.04 (0.60, 1.88)	0.193	1.02 (0.96, 3.00)	0.311
0.1-5.0 kg	1.10 (0.65, 2.76)	0.300	1.14 (0.32, 2.13)	0.405
5.1-10.0 kg	1.20 (0.53, 3.51)	0.451	1.20 (0.72, 2.89)	0.476
>10 kg	1.32 (0.57, 2.77)	0.217	1.18 (0.40, 2.84)	0.647
Ex-smokers vs smokers				
Continuing smokers	Ref		Ref	
Lost weight	0.77 (0.20, 1.77)	0.351	0.66 (0.30, 0.88)	0.015
No weight change	0.81 (0.32, 1.66)	0.201	0.71 (0.44, 0.96)	0.018
0.1-5.0 kg	0.78 (0.34, 1.85)	0.210	0.70 (0.25, 0.90)	0.012
5.1-10.0 kg	0.95 (0.43, 3.03)	0.650	0.79 (0.29, 0.94)	0.021
>10 kg	1.07 (0.58, 2.83)	0.418	0.88 (0.40, 0.92)	0.018
Ex-smokers vs never-smoked				
Never-smoked	Ref		Ref	
Lost BMI	0.88 (0.35, 2.76)	0.219	1.16 (0.60, 2.85)	0.287
No BMI change	1.07 (0.66, 2.79)	0.287	1.12 (0.36, 2.57)	0.138
0.1-2.0 kg m^{-2}	1.16 (0.39, 3.67)	0.614	0.97 (0.55, 2.80)	0.233
>2 kg m^{-2}	1.01 (0.42, 2.96)	0.419	1.17 (0.66, 2.90)	0.258
Ex-smokers vs smokers				
Continuing smokers	Ref		Ref	
Lost BMI	0.79 (0.33, 2.76)	0.415	0.69 (0.28, 0.92)	0.004
No BMI change	0.66 (0.24, 1.73)	0.300	0.73 (0.39, 0.95)	<0.000
0.1-2.0 kg m^{-2}	0.82 (0.45, 3.34)	0.621	0.69 (0.22, 0.88)	<0.000
>2 kg m^{-2}	0.73 (0.23, 1.67)	0.141	0.43 (0.19, 0.81)	0.002

HR (95% CI), hazard ratios (95% confidence interval); BMI, body mass index (Kg/m2); COPD, chronic obstructive pulmonary disease. Adjusted for age, sex, SEIFA, alcohol consumption, dietary pattern, dieting to lose weight, physical activity, employment. The association between smoking status and risk of COPD and mortality was not modified by weight change. The association between weight gain after quitting and risk of COPD and mortality was not modified by duration of

© 2021 Sahle BW et al. *JAMA Network Open.*
